# Supplementary material for: Spanish–speaking validation of the internal corporate social responsibility questionnaire
Source: PLoS One. 2022 Apr 15;17(4):e0266711. doi: 10.1371/journal.pone.0266711 (PMC9012377; doi:10.1371/journal.pone.0266711)
Supplement: S1 Appendix — (DOCX) [file pone.0266711.s002.docx]

**Appendix**

Las siguientes preguntas hacen referencia a varias características que se pueden presentar en su actual trabajo. Usando la escala que se presenta a continuación, indique en qué medida está de acuerdo con cada afirmación (1 = totalmente en desacuerdo, 2 = en desacuerdo, 3 = parcialmente en desacuerdo, 4 = ni de acuerdo, ni en desacuerdo, 5 = parcialmente de acuerdo, 6 = de acuerdo, 7 = totalmente de acuerdo.

| **Spanish** | **English** |
| --- | --- |
| **Estabilidad en el empleo** | **Employment stability** |
| 1. La organización nunca despediría a los empleados de manera discrecional. | 1. The company would never lay off employees at will |
| 1. La organización da a los empleados la sensación de que sus puestos de trabajo son estables y están asegurados en el futuro. | 1. The company gives the employees a feeling that their jobs are also very safe and stable in the future |
| 1. Incluso cuando hay dificultades financieras, la organización hace todo lo posible para asegurar que los empleados permanezcan estables y seguros y no haya despidos forzosos. | 1. Even in difficult financial times, the company is doing everything to ensure the jobs of the employees remain stable and secure and that there will be no compulsory redundancy |
| 1. El empleo en esta organización está casi garantizado. | 1. Employment with the company is almost guaranteed |
| 1. La organización está comprometida con el objetivo de seguridad y estabilidad a largo plazo en el empleo para todos sus trabajadores. | 1. The company is committed to the goal of long - term employment security/stability for all employees |
| 1. Si la organización enfrentara problemas financieros, las reducciones de personal y los despidos masivos serían las últimas opciones por utilizar. | 1. If the company was facing economic problems, employee downsizing and layoffs would be the last option used |
| 1. En general, la organización proporciona a sus trabajadores una alta estabilidad laboral. | 1. Overall, the company provides employees with very high employment stability |
| **Entorno de trabajo** | **Working environment** |
| 1. Las políticas de la organización siempre proporcionan a los empleados un ambiente de trabajo seguro y saludable. | 1. Our organization's policies always provide a safe and healthy working environment for the employees |
| 1. Con el objetivo que los empleados tengan un ambiente de trabajo confortable, la organización sigue siempre los últimos estándares en salud ocupacional (por ejemplo: teclados ergonómicos). | 1. So that employees feel comfortable in their working environment, the company always follows the latest health standards (such as ergonomic keypads). |
| 1. Para asegurar un buen ambiente de trabajo, la organización siempre mantiene los estándares de salud ocupacional e incluso trabaja para mejorarlos. | 1. To ensure a good working environment, the company always maintains the standards of occupational safety and is even developing them further |
| 1. Con el fin de mantener un excelente ambiente de trabajo, la organización siempre analiza y monitorea la salud y los riesgos de seguridad asociados con sus actividades. | 1. The company always analyses and monitors the health and safety risks that are associated with its activities, in order to create an excellent working environment |
| 1. Para asegurar un buen ambiente de trabajo, la organización se esfuerza en todo momento para eliminar los riesgos psicosociales que pueden contribuir a la generación de estrés u otras enfermedades. | 1. To ensure a good working environment, the company strives at all times to remove psychosocial hazards from the workplace that contribute to stress and disease |
| **Desarrollo de habilidades** | **Skills development** |
| 1. La organización apoya a sus trabajadores por medio de programas personalizados de asesoramiento profesional y desarrollo de carrera. | 1. Through career counselling and assistance with career planning the company supports employees in a targeted way |
| 1. La organización apoya a sus trabajadores proporcionándoles siempre el tiempo necesario para aprender nuevas habilidades. | 1. The company supports employees by always providing them with enough time to learn new skills |
| 1. La organización apoya y promueve fuertemente entre sus trabajadores el aprendizaje continuo y el desarrollo de nuevas habilidades. | 1. The company supports and promotes lifelong learning and further development of the employees intensely |
| 1. La organización utiliza para el desarrollo de sus empleados varios métodos y herramientas de aprendizaje (tanto para el desarrollo profesional como personal). | 1. The company uses a blend of many different learning tools and methods for the further development of the employees (with regards to internal and/or external further development) |
| 1. Hay suficientes oportunidades para que los trabajadores mejoren y desarrollen sus habilidades, bien sea por medio de su actual trabajo, o por medio de programas de la organización. | 1. There are more than enough opportunities for employees to improve and develop their skills in their current job or to let their skills be developed by the company |
| 1. En general, la organización estimula a todos sus trabajadores por medio de capacitación y formación en todas las etapas de su experiencia profesional. | 1. Overall, the company encourages all employees at every stage of their professional experience by providing access to vocational training and education |
| **Diversidad** | **Workforce diversity** |
| 1. En la organización hay un buen plan de acción que apoya la igualdad de oportunidades entre todos los trabajadores. | 1. In the company, there is a very good action plan that supports equal opportunities |
| 1. Para asegurar que todos los trabajadores tengan las mismas oportunidades, en la organización existen muy buenas políticas antidiscriminación por cuestiones como género, embarazo, estado civil, discapacidad o raza. | 1. To ensure all employees the same opportunities, in the company there are very good anti-discrimination policies towards issues of gender, pregnancy, marital status, disability and ethnic minorities |
| 1. En términos de igualdad de oportunidades, en la organización existen programas de desarrollo del personal enfocados a mujeres u otras poblaciones vulnerables. | 1. In terms of equal opportunities, in the company there are specialized staff development programmes for women and minorities |
| 1. Con el objetivo de incrementar la igualdad de oportunidades entre sus trabajadores, la organización sobresale por sus buenas políticas orientadas al apoyo de mujeres u otras poblaciones vulnerables. | 1. The company stands for good policies to support women and minorities in order to increase equality of opportunity |
| 1. Con fin de fortalecer la igualdad de oportunidades, la organización trata a todos sus trabajadores de manera justa, equitativa y respetuosa; independientemente de su género, etnia u otras características. | 1. In order to strengthen equality of opportunity, the company treats all employees equally, fairly and with respect, regardless of gender, ethnicity, etc. |
| 1. En general, la organización promueve la igualdad entre sus trabajadores al ofrecer las mismas oportunidades en cualquier situación (independientemente de su género, etnia, etc.). | 1. Overall, the company gives all employees of the organization (regardless of gender, ethnicity, etc.) the same chances and opportunities in every situation and therefore enhances equality of opportunity |
| **Equilibrio entre vida laboral y vida familiar** | **Work-life balance** |
| 1. La organización ofrece a todos los padres y madres programas interesantes de balance vida – trabajo (por ejemplo: instalaciones de cuidado de niños). | 1. With regards to a good balance between work and private life, the company offers all parents attractive programmes (e.g. child care facilities) |
| 1. Con el objetivo de garantizar un buen balance vida – trabajo, la organización ofrece a sus trabajadores opciones flexibles de horario de trabajo. | 1. To ensure a good balance between work and private life, the company offers its employees flexible working time options |
| 1. La organización ayuda a todos sus trabajadores a coordinar su vida personal y laboral de la mejor manera posible, con el objetivo de que se cree un equilibrio saludable entre el trabajo y la vida privada. | 1. The company helps all employees to coordinate their private and professional life in the best possible way, so that a healthy balance between work and private life is created |
| 1. Para promover un buen equilibrio vida – trabajo, la organización ha introducido políticas que prohíben a sus trabajadores tener que laborar horas extras. | 1. To promote a good balance between work and private life, the company has introduced policies that forbid employees from being forced to work overtime |
| 1. Los requerimientos de la organización no interfieren de ninguna manera con el tiempo libre o vida familiar de sus trabajadores, por lo que se garantiza un equilibrio saludable entre el trabajo y la vida privada. | 1. The demands of the company do not interfere with the free time of the employees or their family life to any degree, so that a healthy balance between work and private life is guaranteed |
| 1. Con el objetivo de garantizar un equilibrio saludable entre el trabajo y la vida privada, la organización ofrece una gran flexibilidad con respecto a la hora de inicio y finalización de las actividades laborales. | 1. The company offers great flexibility with respect to the daily work start time and when to go home, so that a healthy balance between work and private life is guaranteed |
| 1. En general, la organización trabaja duro para proporcionar a todos sus trabajadores un muy buen equilibrio entre trabajo y vida privada. | 1. Overall, the company is working hard to provide a very good balance between work and private life for the employees |
| **Involucramiento tangible del empleado** | **Tangible employee involvement** |
| 1. Cuando la organización obtiene buenos resultados financieros, esta comparte parte de ese éxito con sus trabajadores. | 1. If it goes well financially, the company materially shares the organization's success with the employees |
| 1. Si la compañía tiene un buen año financiero, es probable que sus trabajadores puedan participar de esos beneficios. | 1. If it was a successful business year, the employees of the company have very good opportunities for profit sharing |
| 1. Cuando la organización tiene éxito corporativo, esta involucra monetariamente a sus empleados de forma adecuada. | 1. In relation to corporate success, the company materially involves the employees sufficiently |
| 1. Los empleados participan monetariamente del éxito de la organización por medio de bonificaciones anuales. | 1. In the company, the employees are materially involved in the organization's success in a appropriate manner through annual payments |
| 1. La organización sobresale por implementar sistemas de bonificación basados en el desempeño. | 1. The company stands for partly rewarding employees through performance-related bonuses |
| 1. En general la organización comparte su éxito con los trabajadores involucrándolos en algún tipo de compensación económica. | 1. Overall, the company shares its success through involving the employees materially in some manner |
| **Empoderamiento** | **Empowerment** |
| 1. La organización ofrece a sus trabajadores una gran libertad dentro de la cual están facultados para resolver sus problemas laborales de forma independiente. | 1. The company offers its employees great freedom within which they are empowered to solve their problems independently |
| 1. La organización faculta a sus trabajadores para que determinen de forma independiente su propia forma de trabajo que se ajuste a sus límites acordados inicialmente. | 1. The company empowers the employees to determine their own ways of working independently within the agreed boundaries |
| 1. En la organización, los empleados están facultados para corregir de manera independiente los objetivos y tareas asignadas. | 1. In the company, the employees are empowered to independently correct the tasks and goals placed upon them |
| 1. La organización motiva a sus trabajadores a pensar y actuar de forma autónoma e independiente. | 1. The company encourages employees to engage in autonomous and independent thinking and action |
| 1. La organización ofrece a sus trabajadores un alto grado de libertad con respecto al desempeño autónomo de sus tareas. | 1. The company provides employees with a very high degree of autonomy with regards to the independent performance of tasks |
| 1. Los trabajadores de la organización pueden decidir autónomamente como desempeñar sus tareas. | 1. The employees of the company can decide independently how they perform their tasks |
| 1. La organización ofrece a sus trabajadores numerosas oportunidades para que realicen su trabajo en forma independiente. | 1. The company provides the employees with numerous opportunities for independent work |
| 1. En general, los trabajadores de la organización tienen una alta independencia personal en sus responsabilidades y tareas. | 1. Overall, the employees of the company have high personal and independent responsibility in their tasks |
