# Supplementary material for: Spanish–speaking validation of the internal corporate social responsibility questionnaire
Source: PLoS One. 2022 Apr 15;17(4):e0266711. doi: 10.1371/journal.pone.0266711 (PMC9012377; doi:10.1371/journal.pone.0266711)
Supplement: Attachment — Submitted filename: Response to reviewers.docx [file pone.0266711.s004.docx]

All modifications are highlighted in the document

Response to reviewer # 1

**Point # 1:** The authors have carried out all the suggestions so the paper is now ready to be published in this journal.

Thank you for all your previous comments, with your help, our manuscript is now clearer and more focused.

Response to reviewer # 2

**Point # 1:** All my previous comments have been addressed. Please note that following PLOS Data policy, the authors should make all data underlying the findings described in the manuscript fully available.

Thank you for all your previous comments, with your help, our manuscript is now clearer and more focused.

We have included a “supporting information section” that will link the full data set. This section now reads as follow (p. 27):

Supporting information

S1 Dataset. Study sample (.SAV)

In addition, we uploaded the database to the PLOS ONE submission center (we forgot to upload the database during R1)
